# Supplementary material for: Splice-Junction-Based Mapping of Alternative Isoforms in the Human Proteome
Source: Cell Rep. Author manuscript; Available in PMC 2020 Jan 15. (PMC6961840; doi:10.1016/j.celrep.2019.11.026)
Supplement: 2 [file NIHMS1546469-supplement-2.zip › DF1/PXD010154/AdrenalGland/AdrenalGland_10_SYPL1_ETSLHSPSNTSAPHSQGGIPPPTGL.pdf]

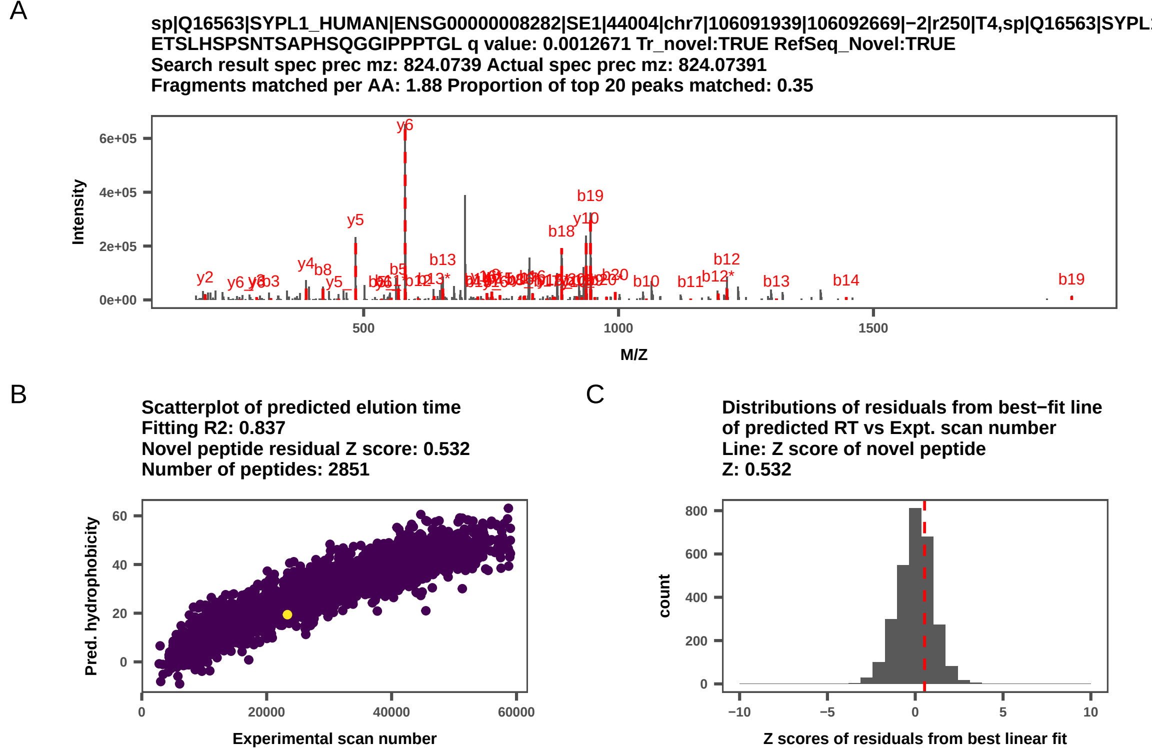

A
sp|Q16563|SYPL1_HUMAN|ENSG00000008282|SE1|44004|chr7|106091939|106092669|−2|r250|T4,sp|Q16563|SYPL
ETSLHSPSNTSAPHSQGGIPPPTGL q value: 0.0012671 Tr_novel:TRUE RefSeq_Novel:TRUE
Search result spec prec mz: 824.0739 Actual spec prec mz: 824.07391
Fragments matched per AA: 1.88 Proportion of top 20 peaks matched: 0.35
Intensity
6e+05
4e+05
2e+05
0e+00
y6
y5
b19
y10
b18
b13
b12
b12*
y4
b8
y2
y3
b3
b5
b13*
b20
b10
b11
b13
b14
b19
500
1000
1500
M/Z
B
Scatterplot of predicted elution time
Fitting R2: 0.837
Novel peptide residual Z score: 0.532
Number of peptides: 2851
Pred. hydrophobicity
60
40
20
0
0
20000
40000
60000
Experimental scan number
C
Distributions of residuals from best−fit line
of predicted RT vs Expt. scan number
Line: Z score of novel peptide
Z: 0.532
count
800
600
400
200
0
−10
−5
0
5
10
Z scores of residuals from best linear fit
